# Moving Beyond Initiative: The Reconceptualization and Measurement of Unethical Pro-organizational Behavior

**DOI:** 10.3389/fpsyg.2021.640107

**Published:** 2021-09-29

**Authors:** Jing Wang, Wei Shi, Guoqin Liu, Li Zhou

**Affiliations:** ^1^School of Management, Zunyi Medical University, Zunyi, China; ^2^School of Labor and Human Resources, Renmin University of China, Beijing, China

**Keywords:** unethical pro-organizational behavior, active UPB, compulsory UPB, measurement, motivation

## Abstract

Despite the fact that unethical pro-organizational behavior (UPB) has become a theoretical topic in the academic field and the fruitful achievements have been explored in the past decade, organizational researches have largely assumed that UPB is an active and voluntary behavior from the perspective of organizational identity and social exchange. In this paper, the authors argue that previous researches have traditionally considered only a very narrow subset of UPB, focusing almost exclusively on extreme voluntary cases which are not reflective of typical UPB. Instead of being primarily voluntary, some typical UPB can be compulsory in nature. We suggest a different look at UPB by contrasting to the so-called “voluntary” activities via compulsory mechanisms in the workplace. Mostly, we are interested in exploring and validating a measurement tool for this behavior. Based on self-determination theory, we argue that such behaviors are a substantial deviation from the original meaning of UPB and thus should be recognized and studied separately. Using six samples, the authors demonstrate the construct validity, reliability, and acceptable psychometric properties of the compulsory UPB scales. Future directions in UPB research are discussed.

## Introduction

What is unethical pro-organizational behavior (UPB)? Scholars often define UPB as “actions that are intended to promote the effective functioning of the organization or its members and violate core societal values, mores, laws, or standards of proper conduct” (Umphress et al., 2010; Umphress and Bingham, 2011). This definition contains two elements. First, UPB is unethical as it violates law, justice or widely held social norms. Second, UPB is unethical behaviors intended to benefit the organization or its members, which reflects a form of contextual performance called “civic virtue”(Castille et al., 2018). It means the definition incorporates the intent for committing the unethical action. “It is carried out consciously, in a discretionary manner, neither ordered by a leader nor part of a formal job description” (Lee et al., 2019; Mishra et al., 2021). Frequently cited examples of UPB include falsifying financial reports, exaggerating the truth about products or services, withholding negative information to customers and clients. In these cases, UPB is primarily assumed as an active and voluntary behavior for the benefit of the organization(Yan et al., 2021), as the actors doing this socially unacceptable behavior by a desire to enhance a firm’s competitive edge, retain valued customers and promote the organization’s interest ultimately (Tian and Peterson, 2016). Are all UPBs voluntary and virtuous? In fact, the voluntary view of UPB is generally not shared by UPB researchers. Instead, some researchers argue that there is an ambiguity in the understanding of individuals’ motivation to engage in UPB (Cheng and Lin, 2019). The notion that UPB represents a somewhat virtuous act only represent an overly simplistic view of one’s UPB motivation (Lee et al., 2019). Some individuals do UPB voluntarily, but others do it just for reasons that they have to Shu (2015), Thau et al. (2015), Tian and Peterson (2016), Ghosh (2017), Lawrence and Kacmar (2017), Zhao and Zhou (2017), Zhang et al. (2017a), Xu and Wang (2020). Unfortunately, current researches have failed to identify a second form of UPB from voluntary UPB, let alone classify different types of UPB.

According to self-determination theory (SDT; Deci and Ryan, 1985), individuals’ intentional behaviors can be voluntary or compulsory. Voluntary behaviors result from autonomous motivation, while compulsory behaviors result from controlled motivation. Organizational scholars have explored lots kinds of motivation for UPB, such as organization identification, transformational leadership, job insecurity, workplace ostracism, high performance demands, performance pressure, psychological empowerment, ethical climates, corporate hypocrisy, and so on (Effelsberg et al., 2014; Shu, 2015; Thau et al., 2015; Tian and Peterson, 2016; Ghosh, 2017; Lawrence and Kacmar, 2017; Zhao and Zhou, 2017; Zhang et al., 2017a). These motivations should lead to different UPBs. However, despite the growth in research focusing on UPB and its inducement, there is a lack of clarity about the full set of UPB types that might differentiate one from the other. Now that we’ve learned a lot about voluntary UPB, what about the compulsory UPB (CUPB)? How should we define and measure it? What is the difference between voluntary and compulsory UPB? In this paper, we will try to find answers for these questions.

The goal of this paper is to move beyond assumptions about UPB and to facilitate future research into this important behavior. We begin by drawing on UPB research and SDT to reconceptualize the UPB construct. We assume that different UPB motivations should lead to different types of UPB, and these UPBs can be classified as active and compulsory UPB. We then develop a compulsory UPB scale by validating its behavioral measures. In so doing, we provide evidence that active UPB and compulsory UPB are different constructs, and we then construct an initial nomological network for compulsory UPB, and establish the criterion-related validity of the compulsory UPB scales. Finally, we chart a path forward for UPB research and discuss new research directions that can result from studying compulsory UPB.

### Unethical Pro-organizational Behavior: Active or Compulsory?

Unethical pro-organizational behavior has traditionally been defined as “actions that are intended to promote the effective functioning of the organization or its members and violate core societal values, mores, laws or standards of proper conduct” (Umphress and Bingham, 2011). Researchers have suggested that our understanding of UPB motivation and type is vague (Cheng and Lin, 2019). Although employees engage in UPB because they believe that doing so will help the company succeed, this is not the only driver of UPB. Employees may engage in UPB to prove that they are valuable organizational members and they deserve to be valued, particularly when they feel their sense of self is threatened (Jachimowicz et al., 2018). Employees may also conduct UPB to avoid the negative consequence caused by the failure to meet the requirement of their organizations (Xu and Wang, 2020). UPB occurs not merely when employees identify with their organizations, but also when they witness their supervisors engaging in similar behaviors and perceive that their supervisors endorse such behaviors (Fehr et al., 2019). According to Cheng and Lin (2019), factors that triggered UPB in previous studies can be divided into three types. First, those aroused individuals to or not to engage in UPB spontaneously; second, factors that exert external pressure and internal drive on an individual to engage in UPB; third, the moral character of an individual or organization. More and more studies began to deviate from the original definition of UPB, pointing to the existence of another UPB type. For example, in their research, Guo et al. (2018) defined UPB as behaviors subject to interpersonal constraints or made out of organizational identification that are intended to promote the effective functioning of the organization or its members and violate core societal values, mores, laws or standards of proper conduct. This definition which has obviously included active and compulsory elements, is different from the definition of Umphress. At the same time, although a large number of empirical studies adopted the UPB definition of Umphress, UPB was regarded as a compulsory behavior in the actual research process. Scholars have found that individuals are not actively and voluntarily engaged in UPB, but have to do so under the pressure of certain factors. Such factors may be the pressure from the organization or the leader, the behavior of colleagues, job insecurity, workplace ostracism, high performance demands, performance pressure, psychological empowerment, ethical climates, corporate hypocrisy and so on (Shu, 2015; Thau et al., 2015; Tian and Peterson, 2016; Ghosh, 2017; Lawrence and Kacmar, 2017; Zhao and Zhou, 2017; Zhang et al., 2017a; Xu and Wang, 2020), which exert external pressure or drive on individuals (Cheng and Lin, 2019). Obviously, in these cases, individuals do not actively engage in UPB out of their organizational identity or positive social exchange. Instead, UPB is more likely to be implemented to avoid certain negative outcomes.

Recent studies suggested that, scholars’ understanding of UPB behavior motivation and behavior type is relatively fuzzy (Cheng and Lin, 2019), and they try to use an UPB construct that only reflects part of its connotation to present the whole picture of UPB, which inevitably affects the accuracy of research conclusions and is not conducive to the comparison of previous research results. Cheng and Lin (2019) pointed out that reconceptualization UPB from the perspective of motivation can solve these problems to some extent. Wang X. C. et al. (2018) also assumed that future research should focus on its nature and underlying motivation of UPB. All in all, these scholars unanimously suggested that the topic of UPB should be re-examined based on motivation theory and the research perspective should be changed.

## Reconceptualization of Unethical Pro-Organizational Behavior on the Base of Self-Determination Theory

As a macroscopic theory of human motivation, SDT has long held its opinion on whether the motivation of an individual to engage in a certain behavior is active or compulsory. It classified the intentional motivation of individuals as autonomous motivation and controlled motivation, and argued that different motivation types reflecting the different psychological process, possessing different causes and consequences, especially leading to two opposite types of behavior: self-determined behavior and non-self-determined behavior.

The most central contribution in SDT is the distinction between autonomous motivation and controlled motivation. Autonomous motivation comprises both intrinsic motivation and the types of extrinsic motivation in which people have identified with its value and have integrated it into their sense of self. When individuals are autonomously motivated, they experience volition and self-choice. Controlled motivation, in contrast, consists of both external regulation and introjected regulation. External regulation means one’s behavior that is a function of external contingencies of reward or punishment, and introjected regulation means the regulation of action that has been partially internalized and is ego-involvement or to avoid of shame, guilt and so on. When individuals are controlling motivated, they feel pressure to think or behave in particular ways. Both autonomous and controlled motivation energize and direct particular behaviors. A number of researches has confirmed that autonomous and controlled motivation can lead to different outcomes, with autonomous motivation leading to greater psychological health and more effective performance, as well as greater long-term persistence.

According to SET, an individual’s intentional behavior should include self-determined behavior and non-self-determined behavior, which are increasingly studied as mutually independent behaviors in recent studies (Amabile, 1993; Grant et al., 2011; Van den Broeck et al., 2013; Strauss et al., 2017). We assume that, as an intentional behavior, UPB should also be differentiated according to the degree of autonomy of motivation.

Scholars argued that proactive behavior is more likely to occur when an individual experiences high autonomous motivation while compulsory behavior is more likely to occur when an individual experiences high controlled motivation (Vigoda-Gadot, 2006; Parker et al., 2010).

Organizational identification which captures the extent to which people define themselves as members of an organization (Haslam et al., 2003; Cornelissen et al., 2007) will help employees integrate UPB’s value into their sense of self. As a result, exerting effort under this circumstance is associated with increased feeling of energy. Therefore, scholars supposed organizational identification to be an important antecedent of UPB (Chen et al., 2016). Meanwhile, variables such as leader–member exchange (LMX) (Lin and Cheng, 2017), organizational commitment (Matherne and Litchfield, 2012), positive employee-organization relations (Wang et al., 2019) can stimulate one’s organizational identity and positive social exchange with the organization, and prompting individuals engagement in UPB with autonomous motivation, easily to cause active UPB. At this time, the individual’s autonomous motivation is in the position of identity regulation on the self-determined continuum. Machiavellians (Castille et al., 2018), confirmed in the previous studies, are keen on engaging in unethical behavior and have a natural interest in the behavior, which will also promote individuals to generate their autonomous motivation to engage in UPB. At this very moment, the individual’s autonomous motivation is in the position of internal motivation on the self-determined continuum.

On the contrary, variables such as high-performance demands (Chen and Liang, 2017), ethical pressure (Tian and Peterson, 2016), and authoritarian leadership (Zhang et al., 2017b; Kang-Hwa and Hung-Yi, 2018) confirmed in previous studies can stimulate one’s controlled motivation in doing UPB, are more likely to lead to compulsory UPB. Now, the controlled motivation locates in the external motivation position of the self-determined continuum. Variables such as psychological entitlement (Sun C. L. et al., 2018; Lee et al., 2019) and overqualification (Chu and Wang, 2019) promote individuals to show excessively high self-evaluation and excessive attention on themselves, which mean controlled motivation of UPB, located in the introjected regulation of the self-determined continuum.

We define UPB as behaviors performed intentionally (actively or compulsorily) by members of an organization in the course of accomplishing tasks and interacting with stakeholders, in order to promote the effective functioning of the organization or its members, but violate core societal values, mores, laws or standards of proper conduct. In this definition, UPB is an intentional behavior as in previous studies. But we emphasize that the intentional UPB should be divided into active and compulsory types. Active UPB are behaviors performed actively and voluntarily by members of an organization in the course of accomplishing tasks and interacting with stakeholders, in order to promote the effective functioning of the organization or its members, but violate core societal values, mores, laws or standards of proper conduct. When autonomously motivated, not only do individuals engage in UPB voluntarily and by the free choice, but also recognize their value and importance. Compulsory UPB are behaviors performed under a certain external pressure by members of an organization in the course of accomplishing tasks and interacting with stakeholders, in order to promote the effective functioning of the organization or its members, but violate core societal values, mores, laws or standards of proper conduct. Individuals are unwilling to but forced to engage in the behavior, do not recognize the value and importance of the behavior. They would not apply the value system guiding the behavior to other areas of life.

To qualify as UPB, behaviors must be intentional behaviors for the sake of the organization or its members. Someone may do anything for the sake of the organization or its members, without any intention to harm or benefit, such as selling a product to consumers without any knowledge of its defects, and this behavior is not included in our definition of UPB. On the contrary, when an employee does know that the product is defective, and sells it to consumers in order to save costs for the company or avoid punishment from leaders. They are all doing UPB. The former is active UPB, and the latter is compulsory UPB.

Whether an action is defined as UPB depends on its motivation rather than its outcome. As long as the initial motivation of non-ethical behavior is for the benefit of the organization, it can be defined as UPB. Employees may sell defective products on their own initiative or under certain pressure, which may reduce customers’ trust and repurchase intention and damage the company’s image. However, the initial purpose of their behaviors is to make profits for the organization, so they all belong to UPB.

Unethical pro-organizational behavior (UPB) should include both business-related UPB aimed at achieving performance goals and relationship-related UPB aimed at maintaining the organization’s image or a lasting relationship with important organizational members. The business-related UPB consists of activities that directly contribute to the realization of the organization”s economic interest objectives. Different from task performance, it is not a task that must be completed by employees explicitly stipulated by the organization. It is also different from job dedication which is one facet of contextual performance. Job dedication includes self-disciplined, motivated acts such as working hard, taking initiative, and following rules to support organizational objectives (Van Scotter and Motowidlo, 1996), while UPB includes behaviors obedience or disobedience the organization rules and may solve job problems actively or compulsorily. Relational-related UPB consists of behaviors dealing with relationships with stakeholders. Different from the concepts of interpersonal relationship taking place in altruistic behavior (Smith et al., 1983), helping behavior (George and Brief, 1992), and interpersonal promotion (Van Scotter and Motowidlo, 1996), relational-related UPB is more involved in dealing with internal and external stakeholders of an organization, such as speaking ill of competitors, coping with external inspection by cheating, ignoring the rights and interests of external stakeholders, etc. Its purpose is not to directly realize economic interests, but to maintain the organization’s image or a lasting relationship with important organizational members.

## Calling for New Unethical Pro-Organizational Behavior Scales

Although the topic of UPB has received great attention in recent 10 years, the development of its scale is relatively slow. Generally speaking, the 6-item scale developed by Umphress et al. (2010) is the most frequently used one in empirical studies, but some scholars point out that certain items of this scale do not fully conform to its definition (Herchen, 2015). A number of empirical studies also show that some items of the scale are not in line with the business practice. For example, many scholars deleted the item “if my organization needed me to, I would give a good recommendation on the behalf of an incompetent employee in the hope that the person will become another organization’s problem instead of my own” (Effelsberg et al., 2014; Shu, 2015; Lin and Cheng, 2016, 2017; Zhang, 2016; Luo and Xv, 2017; Sun Y. B. et al., 2018; Xv et al., 2018; Wang et al., 2019). Although some scholars have tried to develop a suitable scale (Matherne et al., 2018), the universality of the scale is limited because it has not been verified on a large scale and tested in different cultural backgrounds. What’s more, a compulsory UPB scale have not been developed because it’s really a new concept. Therefore, scholars call for future research to develop a more suitable scale (Lin and Cheng, 2017; Wang X. C. et al., 2018; Cheng and Lin, 2019). It is our position that to advance our understanding of UPB, researchers require compulsory measures of UPB.

To solve these problems, we set out to systematically develop and validate a set of UPB scales following advice of Hinkin (1995, 1998). The whole process has been separated into five study phases, each with a special purpose. Refer to Table 1 for an overview of study phases and samples.

**TABLE 1:** Overview of study phases and samples.

Phase description			Samples	
Phase 1—Qualitative Study of UPB Goal: gather examples of UPB to guide item writing and explore latent types of UPB			1 and 2	
Phase 2—Item generation and reduction Goal: generate an item pool and then simplify the items by means of content validity assessment, item analysis, exploratory factor analysis, and confirmatory factor analysis			3, 4, and 5	
Phase 3—Psychometric Properties Goal: assess the internal consistency and inter-scale correlations			5	
Phase 4—Convergent and Discriminant Validity Goal: demonstrate the correlations between UPB scales and related constructs.			5	
Phase 5—Nomological network and criterion-related validity Goal: access relationships between UPB scales and other constructs based on relevant theories.			6	

**Sample description**	***N***	**Date source**		**Location**

Sample 1—Face-to-face interviews	30	Full-time workers		China
Sample 2—Open questionnaire survey	72	Zhihu users		China
Sample 3—Content validity	25	Graduate students		China
Sample 4—Item Analysis	612	Wenjuanxing (full-time workers)		China
Sample 5—Validation sample 1	208	Wenjuanxing (full-time workers)		China
Sample 6—Validation sample 2	265	Field survey (full-time workers)		China

## Phase 1: Qualitative Study of Unethical Pro-Organizational Behavior

We began our research by conducting two qualitative studies, one is an in-depth face-to-face interview, and the other is an open questionnaire survey.

### Materials and Methods

For sample 1, we recruited participants from different sectors, organizations and jobs (*N* = 30; 40% male; mean age = 43.33; mean work experience = 7.23). Face-to-face interviews were conducted with 30 participants. We described what UPB was, and asked participants to provide examples of behaviors that fit to the definition of UPB that they experienced in their daily lives, whether they were engaged in or observed by colleagues, relatives, or friends. Each person’s interview time is limited to about 60 min. In an effort to reduce bias and increase rational decision-making, the term “unethical pro-organizational behavior” was not used in the survey.

For sample 2, we recruited participants from “Zhihu” online community, among whom 72 members received an open questionnaire survey. “Zhihu” is a well-known Chinese online community founded in 2010, and is now open for registration. To June 2018, there had been a total of 6 million paying users in this community, with daily usage frequency exceeding 1 million. On this platform, not only can users share their knowledge, experience and opinion on topics they are interested in, but also follow the topics and users they are interested in. Users can also ask questions on topics they are confused about, or invite other users to answer questions. Within 24 h, users can invite up to 30 people for free. In this study, questions were first asked through “Zhihu” community, and then people related to this topic were invited to answer. Once the questions were answered, the researchers would continue to ask questions, and the respondents were asked to describe the circumstances, reasons and results of this behavior in detail. Five questions were asked and should be answered: “Have you ever witnessed or heard of employees who obeyed public rules for the benefit of the organization?” “Are those employees willing to sacrifice the public interest for the benefit of their own organization or are they forced to do so?” “Have you ever been forced to do something immoral for the benefit of your organization?” “Are human resource workers facing ethical dilemmas?” “Is there anyone who is willing to sacrifice the interests of the masses of society for the benefit of his own organization?” To avoid the defensive psychology of the respondents, we have changed different questioning methods for the same question and cited relevant cases for the convenience of the respondents. In addition, the respondents could also choose to answer anonymously.

### Results

In total, we got 102 detailed responses in this procedure (30 from the in-depth interview and 72 from the open questionnaire). Each response was evaluated by two raters to make sure they met the definition of UPB. The 102 respondents provided UPB cases ranging from 1 to 5 on average. A total of 120 cases were collected, with an average of 1 case per person, including 44 active UPB cases and 76 compulsory UPB cases. The two raters then categorized each case into an appropriate UPB motivation with the help of a researcher-generated list of motivations. Disagreements were solved through rater discussions.

The UPB cases were categorized according to motivation as follows: achieving a win-win situation (3% of UPB cases), repaying one’s organization (4%), for the meaningfulness of the job (5%), duty of work (5%), the consistent interests of the organization and employees (8%), leadership identification (1%), organizational identification (6%), requirements of leaders (3%), for wages (22%), workplace ostracism (3%), performance pressure (17%), job insecurity (5%), ethical climate (4%), career development (5%), to win the recognition and reward of the leader (5%), self-recognition (1%), with no responsibility (1.5%), to avoid feeling shamed(1.5%).

### Discussion

Although it is often assumed that UPB is solely voluntary, our evidence indicated that individuals had engaged in both voluntary and compulsory UPB. The relative frequency of compulsory UPB was higher than voluntary UPB. Overall, respondents reported engaging in UPB to satisfy all kinds of individual needs, including pressure from others. In summary, we should take a more balance view on UPB than a purely voluntary view.

## Phase 2: Item Generation and Reduction

Based on qualitative study and combined with previous research results, this paper constructed a compulsory UPB scale with 21 items. See Appendix A for the original 21 items.

### Substantive Validity Assessment

Following the advice of Hinkin (1998) and the practice of Brady et al. (2017), we used an item-sort task to assess the substantive validity of the scale (Anderson and Gerbing, 1991). The primary goal was to retain items with substantive validity and eliminate items without it.

### Participants and Procedure

For sample 3, participants were graduate students majoring in psychology (*N* = 25; 48% female; mean age = 22.52 years). Participants were given the list of 21 compulsory UPB items and eight construct definitions. Participants were then asked to choose the most appropriate construct for each item. The eight constructs were UPB, organizational citizenship behavior, compulsory citizenship behavior, counterproductive work behavior, organization misbehavior, workplace deviance, pro-social rule breaking, Illegal corporate behavior.

### Results

Following the procedure of Anderson and Gerbing (1991), we calculated the substantive-validity coefficient (C_SV_) and the critical value of C_SV_ (−C_SV_). Then, C_SV_ was compared to the calculated C_SV_ value. “If an item’s C_SV_ value is equal or greater than the −C_SV,_ then it should be retained for further analysis” (Howard and Melloy, 2016), or it should be deleted. Finally, 4 items were eliminated and 17 items were retained. See Appendix B for the 17 items after content validity assessment.

### Item Analysis

We next set out to do item analysis to eliminate items further with the help of sample 4 and 5. Sample 4 was used for an exploratory factor analysis (EFA), and sample 5 was used for a confirmatory factor analysis (CFA). Hinkin (1995, 1998) pointed out that most constructs can be measured by four to six items. Our aim during this procedure was to further reduce our items.

### Participants and Procedure

For sample 4, an online crowdsourcing platform in mainland China named Wenjuanxing was used for an online survey, which provided functions equivalent to Amazon Mechanical Turk (*N* = 612; 55.4% female; mean age = 23.65; mean work experience = 7.32 years; 71.2% participants holding a bachelor degree).

For sample 5, Wenjuanxing was used to recruit full-time workers for an online survey (*N* = 208; 54.3% male; mean age = 31.32; mean work experience = 6.06 years; 73.9% participants holding a bachelor degree).

### Results and Discussion

Before the implementation of EFA, we followed the process of Brady et al. (2017) to eliminating items which were deemed to “not be as conceptually important or which were statistically redundant to other items.” After this, 1 item was deleted. Finally, a scale with 16 items was formed (see Appendix C for the 16 items left.).

We performed an EFA (maximum likelihood with promax rotation) on the 16 compulsory UPB items. Two factors with eigenvalues greater than 1.0 were identified (total variance extracted = 64.64%). As expected, all items clearly loaded onto the 2 intend factors (factor loading = 0.63–0.88): business-oriented compulsory UPB (CBOU), relationship-oriented compulsory UPB(CROU), with four items for the former and three items for the later (see Table 2).

**TABLE 2:** Results of EFA.

Item	CBOU	CROU
*In the last month, how often have you*…		
Had to misrepresent the truth to make your organization look good.	**0.80**	0.20
Had to ignore the rights and interests of people outside the organization under certain pressures.	**0.79**	0.06
Had to take some cheating measures to help the organization pass the external inspection.	**0.77**	0.18
Had to depreciate competitors for the benefit of the organization under certain pressures.	**0.63**	0.36
Had to highlight the advantages of your product or service and avoided the disadvantages under certain pressure.	0.11	**0.88**
Had to weaken the shortcomings of the product or service for the benefit of your organization under certain pressures.	0.22	**0.82**
Had to conceal information that is not conducive to the sales of your products under certain pressures.	0.22	**0.73**

*CBOU, business-oriented compulsory UPB; CROU, relationship-oriented compulsory UPB. The highest loadings are shown in bold.*

A CFA was then performed on the remaining items to access model fit. Inspection of the residuals showed that the seven items have good model fit, χ^2^/df = 2.37, comparative fit index (CFI) = 0.90, standardized root-mean-square residual (SRMR) = 0.04 (see Table 3).

**TABLE 3:** Comparison results of confirmatory factor analysis model.

^*Model*^	^χ^ ^2^	^*df*^	^*RMSEA*^	^*CFI*^	^*TLI*^
^*M1*^	^83.98^	^14^	^0.16^	^0.87^	^0.80^
^*M2*^	^23.58^	^13^	^0.06^	^0.98^	^0.97^

*M1, single-factor model; M2, two-factor model; M3, three-factor model; M4, four-factor model.*

With these evidences, the seven items were deemed to be finalized. So, the CUPB scale consists of two scales: business-oriented compulsory UPB and relationship-oriented compulsory UPB. The final UPB scales are shown in Appendix D (English version) and E (Chinese version).

## Phase 3: Psychometric Properties

We next evaluated the psychometric properties of the UPB scales, which meant that the internal consistency and inter-scale correlations for the compulsory UPB scale should be estimated.

### Participants

We used sample 5 again in this step.

### Results

#### Internal Consistency

Reliabilities were all good. The reliability of the compulsory UPB scale was 0.85, and the reliability of the CBOU and CROU were 0.80 and 0.80.

#### Inter-Scale Correlations of Compulsory Unethical Pro-organizational Behavior

Mean correlation between the two compulsory UPB scales was positive and significant (CBOU and CROU: *r* = 0.57, *p* < 0.01). The correlation was not too high to differentiate one from the other.

### Discussion

Evidence confirmed our two-factor compulsory UPB scale again, with each factor corresponding to a single UPB scale. Reliabilities for each of the compulsory UPB scales were perfect and the UPB inter-scale correlation was positive and significant. Overall, results showed the compulsory UPB scale possesses acceptable psychometric properties.

## Phase 4: Convergent and Discriminant Validity

We used sample 5 to assess the convergent and discriminant validity of the compulsory UPB scale with two different methods.

To access convergent validity, we tested the CUPB scale against amorality (a dimension of Machiavellian Personality; Dahling et al., 2009), as Mesdaghinia et al. (2018) pointed out that UPB was positively correlated with yet distinguishable from a stable amoral personality. We expected the positive correlation between CUPB and amorality to be significant, but not so high that the scales measure the same construct.

As discussed in this paper, UPB has traditionally been seen as an active behavior. In this study, we have argued that some UPB does not fit this conceptualization. Compulsory UPB may exit in workplace. We therefore try to establish the discriminant validity of CUPB by demonstrating that CUPB is not an autonomous behavior. We expected compulsory UPB to be negatively correlated with, yet distinguishable from job autonomy and citizenship behaviors. That is because individuals with job autonomy can determine his own behavior. But the actors of compulsory UPB without job autonomy will not have this right, which leads to the negative correlations between compulsory UPB and job autonomy. UPB should also be distinguishable from citizenship behaviors which seek to benefit the organization in ethical ways.

### Measures

***Compulsory UPB(CUPB)*** was measured separately with the seven-item scale developed in this study. Sample items was “had to highlight the advantages of your product or service and avoided the disadvantages under certain pressure.” The internal consistency estimating for the scale was 0.84.

***Job autonomy*** was assessed with the three-item scale developed by Spreitzer (1995) (α = 0.89). Sample item was, “I have significant autonomy in determining how I do my job.” Participants responded to the items on a 7-point scale ranging from *strongly disagree* (1) to *strongly agree* (7).

***Organizational citizenship behaviors*** were measured with the eight-item scale which directed to the organization(OCBO) developed by Lee and Allen (2002) (α = 0.83). Sample item was “Attending functions that are not required but that help the organizational image.” Participants were asked to indicate, using 7-point scales (1 = never, 7 = always), how often they engaged in these behaviors.

***Amorality*** was assessed with the five-item scale developed by Dahling et al. (2009) (α = 0.76). Sample item was “I believe that lying is necessary to maintain a competitive advantage over others.” Participants responded to the items on a 7-point scale ranging from *strongly disagree* (1) to *strongly agree* (7).

### Results

As predicted, there was a positive correlation between CUPB and amorality (*r* = 0.46, *p* < 0.01). What’s more, both job autonomy and OCBO had significantly negative correlation with compulsory UPB (*r* = –0.09, *p* < 0.05; *r* = –0.16, *p* < 0.01). The results provided initial evidence in support of the convergent validity and the distinctness of the CUPB scale.

Next, CFAs were performed for discriminant validity test. As shown in Table 4, a four-factor model was a better model fit than other models. And this demonstrated the distinctness of the four scales.

**TABLE 4:** Comparison results of confirmatory factor analysis model.

Model	^χ^ ^2^	df	TLI	CFI	RMSEA
Model 1 Four-factor model	562.19	129	0.92	0.91	0.07
Model 2 Three-factor model (O + A, C, M)	994.57	132	0.76	0.79	0.10
Model 3 Three-factor mode l (C + M, O, A)	846.52	132	0.80	0.83	0.09
Model 4 Three-factor mode l (C + O, A, M)	1265.01	132	0.68	0.72	0.12
Model 5 Three-factor model (C + A, O, M)	1694.58	132	0.56	0.62	0.14
Model 6 Two-factor model (C + A, O + M)	2327.93	134	0.39	0.47	0.16
Model 8 Two-factor model (C + O, M + A)	2313.58	134	0.32	0.41	0.23
Model 9 One-factor model	2599.09	135	0.32	0.40	0.40

*C, CUPB; O, OCBO; A, job autonomy; M, amorality.*

Further, the Fornell and Larcker (1981) test of convergent and discriminant validity showed that the CUPB scales were all good as the AVE of all the CUPB scales were greater than 0.5 (AVE: CBOU = 0.65, CROU = 0.72). The AVE by both factors was always greater than the squared correlation between the constructs (smallest AVE for other scales = 0.65; largest *r*^2^ = 0.21). These results provided evidence of convergent and discriminant validity again.

### Discussion

In this phase, the convergent validity of the CUPB scales was established as CUPB was positively correlated with amorality. The discriminant validity of the CUPB scales was established using measures of OCBO and job autonomy. Although it has been traditionally assumed that UPB is an active behavior, we demonstrated that some UPBs (e.g., CUPB) are distinct from measures of OCBO and job autonomy which involves initiative. Overall, all evidence supported the convergent and discriminant validity of the UPB scales.

## Phase 5: Nomological Network and Criterion-Related Validity

### Nomological Network

#### Affective Commitment

Affective commitment means “an emotional attachment to, identification with, and involvement in the organization” (Meyer and Allen, 1991). A variety of studies have proposed that affective commitment and organization identification are related. Organizational commitment includes the statement that individuals with high organizational commitment will exert considerable effort on behalf of the organization (Porter et al., 1974). Cullinan et al. (2008) suggested that individuals with higher levels of affective commitment are less likely to engage in organization-harm unethical behaviors which harm the organization. On the contrary, in order to maintain a strong sense of identity with the organization, they will try their best to avoid harming the organization, and are even willing to falsify financial information and implement other unethical behaviors in order to achieve the goals of the organization (Matherne and Litchfield, 2012). As Fernet et al. (2012) argued that affective commitment was positively correlated with autonomous motivation and negatively correlated with controlled motivation, strong organizational identity and emotional commitment motivate individuals to take the initiative to act in a way that is beneficial to the organization. Individuals with high affective commitment will be less likely to engage in CUPB.

**Hypothesis 1:** CUPB will be negatively related to affective commitment.

#### Leader-Member Exchange

The main tenant of LMX is that, through different types of exchanges, leaders differentiate their ways of treating their followers. According to LMX, the higher quality of the relationship that develops between a leader and his follower is predictive of lots of positive performance-related and attitudinal outcomes (Gerstner and Day, 1997). In a high-quality exchange relationship, the exchange of leaders and employees will go beyond the scope of work and develop to a higher level of relationship quality (Graen and Uhl-Bien, 1995). The leader may permit the followers job autonomy and a broader scope of decision-making, show their trust, give more career opportunities, while followers may maintain a positive motivation to repay their leaders, show more work effort to support their leaders, improve their creativity and performance, as also as engage in a certain risk in favor of their leaders (Johnson and Umphress, 2019). Cai et al. (2018) recognized that, direct interpersonal interactions and relationships, such as LMX could be predictor of proactive employee behavior. When employees establish a high-quality exchange relationship with their leaders, they will feel no pressure to engage in work behaviors (Chambel et al., 2015). On the contrary, if employees establish a low-quality exchange relationship with their leaders, they always feel compelled to engage in work behaviors. Accordingly, we advanced the following hypothesis.

**Hypothesis 2:** CUPB will be negatively related to LMX.

#### Perceived Organizational Support

Perceived organizational support (POS) means employees develop a general perception concerning the extent to which the organization values their contributions and cares about their well-being (Kurtessis et al., 2017). According to the norm of reciprocity, POS should lead to a felt obligation to help the organization, such as engaging in greater job-related efforts, enhancing in-role job performance and extra-role performance helpful to the organization. Meta-analysis showed that, POS was positively related to trust in the organization, organizational identification, affective commitment, job involvement, job satisfaction, self-efficacy, and so on (Kurtessis et al., 2017). The high POS employees will do more pro-organizational behavior in return for the favor of the organization regardless of whether the behavior violates ethical standards (Luo and Xv, 2017). But employees with low POS may not do everything for the organizations if not ordered or compelled by their leaders and organizations.

**Hypothesis 3:** CUPB will be negatively related to POS.

#### Job Insecurity

Job insecurity means that individuals worry about becoming jobless and this feeling will threaten a person’s social identity as an employed person which in turn will affect well-being and job performance. Persons who feel higher levels of job insecurity are more likely to report a weaker organizational identity (Selenko et al., 2017). And someone who has a weaker organizational identity will be less likely to show organizational member proactivity, which entails future-directed behavior aimed to increase the befit of the organization. Some researchers do have found that employees will deal with the bad feeling of job insecurity by working hard (Armstrong-Stassen, 2006), impression management (Huang, Hua et al., 2013), and engaging in behaviors that are unethical but pro-organizational (Ghosh, 2017). According to SDT, job insecurity means external contingencies which make individuals experience pressure to think, feel or behave in particular ways. And these particular ways are non-self-determined ways.

**Hypothesis 4:** CUPB will be positively related to job insecurity.

### Criterion-Related Validity

#### Guilt

Defined as emotion-based regret associated with a negative event, guilt is an emotion closely linked to ethical and unethical behavior. Guilt prompts individuals to internalize responsibility for behavior that violates his personal ethical standards). Tang et al. (2020) found that, after engaging in volitional UPB, sales agents always felt guilt. Not only is guilt a consequence of active behavior, but also a consequence of compulsory behavior. For example, Umphress et al. (2010) argued that guilt is probable a consequence that results from UPB, an unethical behavior someone does it voluntarily. On the contrary, Mesdaghinia et al. (2018) also confirmed that when some moral individuals who neither have the option of changing employer nor are able to make a change in the situation may experience strong guilt emotion.

**Hypothesis 5:** CUPB will be positively related to guilt.

#### Turnover Intention

According to SDT, motivation is the critical driver of attitude and behavior. Incented by the autonomous motivation, individuals will take part in an activity for enjoyment. But if incented by the controlled motivation, they seek for purposes beyond work. As Mesdaghinia et al. (2018) argued, when individuals did UPB under the pressure of their leaders they would leave their organization. So, we expect compulsory UPB, which is influenced by the situation, to be positively correlated with turnover.

**Hypothesis 6:** CUPB will be positively related to turnover intention.

### Measures

***Affective commitment*** was assessed with the six-item scale developed by Allen and Meyer (1990), and the internal consistency estimated for the scale was 0.84. Sample item was, “This organization has a great deal of personal meaning for me.” Respondents used a 5-point scale ranging from *strongly disagree* (1) to *strongly agree* (5) to respond to those items.

***Leader–member exchange*** was assessed with the seven-item scale developed by Graen and Uhl-Bien (1995), and the internal consistency estimated for the scale was 0.86. Sample item was, “my leader understands my job problems and needs.” Respondents used a 5-point scale ranging from *strongly disagree* (1) to *strongly agree* (5) to respond to those items.

***Perceived organizational support*** was assessed with the six-item scale developed by Eisenberger et al. (2001), and the internal consistency estimated for the scale was 0.90. Sample item was, “The organization is willing to help me if I need a special favor.” Respondents used a 5-point scale ranging from *strongly disagree* (1) to *strongly agree* (5) to respond to those items.

***Job insecurity*** was assessed with the four-item scale developed by Vander Elst et al. (2014), and the internal consistency estimated for the scale was 0.81. Sample item was, “chances are that I will soon lose my job.” Respondents used a 5-point scale ranging from *strongly disagree* (1) to *strongly agree* (5) to respond to those items.

***Guilt*** was measured using six items from PANAS-X Watson and Clark (1988), and the internal consistency estimated for the scale was 0.79. Participants were asked to rate their guilt over the past 30 days.

***Turnover intention*** was assessed with three items developed by Konovsky and Cropanzano (1991) (α = 0.91). Sample item was, “How likely is it that you will look for a job outside of this organization during the next year?” Respondents used a 7-point scale ranging from *very unlikely* (1) to *very likely* (7) to respond to those items.

***Compulsory UPB(CUPB)*** was measured separately with the seven-item scale developed in this study.

### Participants

We used sample 6 in this step. Sample 6 was an independent, multi-wave data sample recruited with the help of the authors’ friends. All the participants are full-time workers. A total of 265 participants completed the first wave (72% response rate; 43% female; mean age = 34.39, *SD* = 11.10; organization tenure = 3.78, *SD* = 4.23). One week later, 233 participants completed the second wave (86% retention rate). Participant education level was varied (high school = 12%; university or college = 73%; master’s degree = 15%).

### Results

Correlations and reliabilities for Sample 6 are shown in Table 5.

**TABLE 5:** Descriptive statistics, correlations, and alpha reliabilities for sample 6.

Variable	*M*	*SD*	1	2	3	4	5	6	7
(1) CUPB	3.36	0.86	**0.81**						
(2) AC	4.07	0.92	–0.13**	**0.84**					
(3) LMX	4.10	0.83	–0.18**	0.52**	**0.86**				
(4) POS	4.08	0.90	–0.17**	0.67**	0.62**	**0.90**			
(5) JI	2.31	0.85	0.13**	–0.51**	–0.40**	–0.51**	**0.81**		
(6) Guilt	1.92	0.66	0.14**	–0.13**	–0.23**	–0.16**	0.30**	**0.79**	
(7) Turnover	3.44	1.33	0.13**	–0.55**	–0.41**	–0.57**	0.54**	0.23**	**0.91**

*n* = 233; Cronbach’s alpha reliabilities are on the diagonal in bold. CUPB, compulsory UPB; AC, affective commitment; JI, job insecurity. *p < 0.05; **p < 0.01; ***p < 0.001.

#### Affective Commitment

As predicted in H1, the compulsory UPB scale was negatively related to affective commitment (*r* = –0.13, *p* < 0.01).

##### Leader–member exchange

As predicted in H2, CUPB was negatively related to LMX (*r* = –0.18, *p* < 0.01).

##### Perceived organizational support

As predicted in H3, CUPB was negatively related to POS (*r* = –0.17, *p* < 0.01).

##### Job insecurity

As predicted in H4, there was a significant positive correlation between CUPB and job insecurity (*r* = 0.13, *p* < 0.01).

##### Guilt

As predicted in H5, CUPB was positively related to guilt (*r* = 0.14, *p* < 0.01).

##### Turnover intention

As predicted in H6, CUPB was positively related to turnover intention (*r* = 0.13, *p* < 0.01).

### Discussion

Results supported the nomological network of CUPB, showing that CUPB is related to a variety of variables which have previously been theorized to relate to UPB, including affective commitment, LMX, POS, and job insecurity. Relations were also demonstrated between CUPB and two criterion variables, including guilt and turnover intention. Overall, all evidences supported the nomological network and criterion-related validity of the CUPB scale.

## Findings

Using six samples, we reconceptualized the UPB construct, and then proposed the CUPB concept and validated the CUPB scale in this study. We employed both qualitative and quantitative methods to identify the theoretical structure of CUPB, and found that CUPB was made up of CBOU and CROU. We developed a 7-item CUPB scale and established the initial reliability and validity of this new scale. As was shown in this study, CUPB include business-oriented CUPB aimed at achieving performance goals and relationship-oriented CUPB aimed at maintaining the organization’s image or a lasting relationship with important organizational members. The positive correlation between CUPB and amorality (*r* = 0.46, *p* < 0.05) provided evidence of the convergent validity of the UPB scales; while the inverse correlation relationship between CUPB and job autonomy as well as OCBO (job autonomy: *r* = –0.09, *p* < 0.05; OCBO: *r* = –0.16, *p* < 0.01) supported the distinctness of the UPB scales. Further, the value of AVE also supported the convergent and discriminant validity of our UPB scales for the two CUPB scales’ AVE were greater than 0.5 (AVE: CBOU = 0.65, CROU = 0.72), and these values were all greater than the square of the correlation coefficient between the concerned constructs (largest *r*^2^ = 0.21). Tests of the UPB nomological network and criterion-related validity were consistent with previous UPB theory, and showed that the UPB scales were related to important organizational variables and processes such as affective commitment, LMX, POS, job insecurity, guilt and turnover intention.

## General Discussion

Despite previous theory and evidence disciplined that UPB was an intentionally voluntary behavior that individuals did it out of best wishes for the company (Umphress et al., 2010; Umphress and Bingham, 2011), it ignored an important type of UPB under some kind of pressure, such as commands or instructions from leaders or organizations, ethical climate, performance pressure, threats of losing one’s job. This represent a serious ambiguity in the way that UPB is conceptualized and studied. We have argued that this ambiguity may have led to vague or wrong conclusions. Although some cases of UPB may be active, our qualitative evidence suggested that there were also compulsory cases of UPB.

To facilitate future UPB research, we reconceptualized the UPB construct, and validated CUPB scale. With the help of six samples, including multi-wave data, we demonstrated that the CUPB scale was valid and reliable measures of CUPB. Besides, evidence showed that the model fits of CUPB scale was good. Discriminant validity tests showed that the CUPB scale was different from job autonomy and OCBO; while the convergent validity tests showed that the CUPB scale shared a lot in common with amorality. Tests of the CUPB nomological network and criterion-related validity showed that the CUPB scale was related to critical organizational variables. These findings were consistent with previous proactive behaviors studies, as scholars argued that proactive behaviors were distinct from more passive behaviors (Parker and Collins, 2010).

### Theoretical Implications

This study provides several contributions to the current theory. First, the primary contribution of this study is that it is the first to propose the concept of “compulsory UPB.” Can we put all UPB in one basket of voluntary UPB, elsewhere defined as “blind devotion” (Wang H. Y. et al., 2018)? What happens when employees’ good will is misused by his/her organization or supervisor? Our paper suggests that not all UPB can be put in one basket of active behavior. Formal and informal coercive actions by leaders or the organizational environment can result in compulsory UPB that are involuntary and ultimately destructive. We urge researchers to move beyond voluntary assumptions about UPB and to take a balanced view of the behavior. This will allow us to recognize both voluntary and compulsory aspects of UPB. Our intention is not to argue that UPB is always negative, but rather that UPB is a complex behavior which is typically not as active as people assume. The general phenomenon of UPB can thus be interpreted along a continuum with two ends. The first represents voluntary activities that are aimed at benefiting the organization. The second represents compulsory activities forced by others to invest effort beyond one’s duties. This second end is a negative deviation from the first one, which against one’s good will, and may result in harmful outcomes.

Second, an innovative strand of the present work is our use of self- determination theory to distinguish UPB types, in contrast to past research that has described the active and compulsory behavior types through reference to coercive persuasion theory (Vigoda-Gadot, 2006). Specially, our research based on SDT, proposes the concept of “active UPB” and “compulsory UPB” to denote different UPB activities. We further explored the dimensions of the CUPB scale to find that it was consist of business-oriented aspect and relationship-oriented aspect.

Third, an important contribution of our research is the extension of the nomological network of UPB. Beyond the four types nomological network of UPB summarized by Yan et al. (2021), we found that turnover intention, guilt and LMX were all related to CUPB. So, another type named emotional variables should be added to the nomological network of UPB.

### Practical Implications

Beyond the theoretical implications that our study has for the future development of the concept of UPB, it may also have some practical implications. First, I propose that the concept of UPB involved in current researches is voluntary UPB, compulsory UPB is also quite prevalent in many organizations. For example, the pressure to promote task performance by any kind of means frequently heard in sales company. Thus, a major practical implication of this paper is that managers should come to a clear agreement with employees about ethical means to achieve organization goals. This can be achieved by improving the moral level of leaders (Lian et al., 2020; Schuh et al., 2021), creating an ethical climate (Zhao and Zhou, 2017), providing adequate support system to employees who are under pressure (Ghosh, 2017) and so on.

Another practical implication of this paper is that employees should be encouraged to speak up when they feel they are forced to do unethical things for the benefit of the organization. This can be achieved by strict supervision mechanism, improved communication channels, or by mutual negotiations among members of the organization.

### Limitations

There are some limitations with our studies that should be considered.

First, we haven’t performed a cross-cultural measurement invariance test. The conclusion obtained in the Chinese context may not be suitable for other cultural contexts. For example, previous studies have found that UPB may take different forms in different countries (Effelsberg et al., 2014; Shu, 2015; Wang et al., 2019).

Second, we used only guilt and turnover intention as criterion variables. We encourage future researches to include other important outcomes variables, such as organizational performance, wellbeing, work-related stresses.

Another potential limitation is the use of a single-wave and single-source sample in the construct validation (i.e., sample 5), which may lead to common method bias. For example, affective commitment, LMX, POS and job insecurity were self-rated by employees, and this may lead to common method bias. We did try to reduce this common method variance by collecting multi-wave data in sample 6. In all cases, evidence demonstrated that the UPB scales had acceptable psychometric properties.

### Future Research Into Unethical Pro-organizational Behavior

First, too much attention has been payed to the determinants of UPB while the outcome of it was neglected comparatively. The CUPB scale developed in this research can be used as a tool to verify whether active UPB and compulsory UPB can lead to different outcomes. Previous studies have demonstrated that a workplace behavior of the doer, active or compulsory, is a decisive factor, beneficial or not to the individuals career success and work well-being (Vigoda-Gadot, 2007; Duan et al., 2019). Future studies can evaluate this by considering the nature of UPB employees tend to enact.

Second, although Umphress et al. (2010) have constructed the theoretical model of UPB 10 years ago, there have been no researchers to verify this model. Future research should modify the model to make an active and compulsory UPB to test their propositions.

Finally, future research is likely to get benefit by extrapolating UPB to broader levels of analysis, particularly at team and organization levels. Previous studies demonstrated that teams sharing a common sense of performance pressure may develop a climate of high self-interest and self-protection. They may adopt a team-level UPB as a result. In addition, as individuals are often nested within teams, an individual UPB may easily affect the ethical behavior of team and organization. So, we encourage future researchers to pay attention to team and organization level UPB, especially their active and compulsory type.

## Data Availability Statement

The original contributions presented in the study are included in the article/Supplementary Material, further inquiries can be directed to the corresponding author/s.

## Author Contributions

JW was responsible for concept construction, research design and data analysis, while WS was responsible for the acquisition of data. GL revised the important intellectual content of the article, while LZ download articles and edited the manuscript. All the authors contributed to the article and approved the submitted version.

## Conflict of Interest

The authors declare that the research was conducted in the absence of any commercial or financial relationships that could be construed as a potential conflict of interest.

## Publisher’s Note

All claims expressed in this article are solely those of the authors and do not necessarily represent those of their affiliated organizations, or those of the publisher, the editors and the reviewers. Any product that may be evaluated in this article, or claim that may be made by its manufacturer, is not guaranteed or endorsed by the publisher.
