# Supplementary material for: Moving Beyond Initiative: The Reconceptualization and Measurement of Unethical Pro-organizational Behavior
Source: Front Psychol. 2021 Sep 29;12:640107. doi: 10.3389/fpsyg.2021.640107 (PMC8511457; doi:10.3389/fpsyg.2021.640107)
Supplement: Supplementary file 1 [file Table_1.pdf]

Supplementary Material

Appendix A

The original 42 items for content validity assessment

INSTRUCTIONS: The following questions are about workplace behaviors you may have come across. In the last month, how often have you···(Items are evaluated on a 7-point response scale for 1=never, 2=once a month, 3=2-3 times a month, 4=once a week, 5=2-3 times a week, 6=once a day, 7=more than once a day).

Compulsory UPB

Items	Item source	SDT reflection
1. had to gather information on competitors to benefit your organization.	Qualitative study	Controlled motivation
2. had to conceal information that is not conducive to the sales of your products under certain pressures.	Umphress et al. (2010)	Controlled motivation
3. had to highlight the advantages of your product or service and avoided the disadvantages under certain pressure.	Qualitative study	Controlled motivation
4. had to weaken the shortcomings of the product or service for the benefit of your organization under certain pressures.	Qualitative study	Controlled motivation
5. had to accept decisions that may offend the interests of others outside the organization.	Qualitative study	Controlled motivation
6. had to exaggerate the truth to promote the cooperation between the partner and your organization.	Qualitative study	Controlled motivation
7. had to depreciate competitors for the benefit of the organization under certain pressures.	Qualitative Study	Controlled motivation
8. had to turn a blind eye to our organization's behavior that offend the interests of others outside the organization.	Qualitative Study	Controlled motivation
9. had to conceal information from the public that could be damaging to your organization.	Umphress et al. (2010)	Controlled motivation
10. had to misrepresent the truth to make your organization look good.	Umphress et al. (2010)	Controlled motivation
11. had to keep silent about actions that are beneficial to the organization but contrary to the general interest of society.	Qualitative study	Controlled motivation
12. had to misrepresent the truth to make your organization look good.	Umphress et al. (2010)	Controlled motivation
13. had to take some cheating measures to help the organization pass the external inspection.	Matherne & Litchfield(2012), Qualitative study	Controlled motivation

14. had to exaggerate data or information that is good for the organization under certain pressures.	Umphress et al. (2010)	Controlled motivation
15. had to ignore the rights and interests of people outside the organization under certain pressures.	Matherne & Litchfield(2012), Qualitative Study	Controlled motivation
16. had to recommend customers to buy products that are not suitable for them to help your organization.	Qualitative study	Controlled motivation
17. had to offer special benefits to partners in order to facilitate the work.	Qualitative study	Controlled motivation
18. had to deflect or avoid topics that are not favorable to your organization.	Qualitative study	Controlled motivation
19. had to accept special interests presented by partners to facilitate the work.	Qualitative study	Controlled motivation
20. had to withhold issuing a refund to a customer or client accidentally overcharged.	Umphress et al. (2010)	Controlled motivation
21. had to withhold to customers the discounts he/she can get form your company.	Qualitative study	Controlled motivation

Appendix B

The 34 items after content validity assessment

INSTRUCTIONS: The following questions are about workplace behaviors you may have come across. In the last month, how often have you…(Items are evaluated on a 7-point response scale for 1=never, 2=once a month, 3=2-3 times a month, 4=once a week, 5=2-3 times a week, 6=once a day, 7=more than once a day).

Compulsory UPB

1. had to gather information on competitors to benefit your organization.
2. had to conceal information that is not conducive to the sales of your products under certain pressures.
3. had to highlight the advantages of your product or service and avoided the disadvantages under certain pressure.
4. had to weaken the shortcomings of the product or service for the benefit of your organization under certain pressures.
5. had to accept decisions that may offend the interests of others outside the organization.
6. had to exaggerate the truth to promote the cooperation between the partner and your organization.
7. had to depreciate competitors for the benefit of the organization under certain pressures.
8. had to turn a blind eye to our organization's behavior that offend the interests of others outside the organization.
9. had to conceal information from the public that could be damaging to your organization.
10. had to misrepresent the truth to make your organization look good.
11. had to misrepresent the truth to make your organization look good.
12. had to take some cheating measures to help the organization pass the external inspection.
13. had to exaggerate data or information that is good for the organization under certain pressures.
14. had to ignore the rights and interests of people outside the organization under certain pressures.
15. had to recommend customers to buy products that are not suitable for them to help your organization.
16. had to deflect or avoid topics that are not favorable to your organization.
17. had to withhold to customers the discounts he/she can get form your company.

Appendix C

The 32 items after Item Analysis

INSTRUCTIONS: The following questions are about workplace behaviors you may have come across. In the last month, how often have you… (Items are evaluated on a 7-point response scale for 1=never, 2=once a month, 3=2-3 times a month, 4=once a week, 5=2-3 times a week, 6=once a day, 7=more than once a day).

Compulsory UPB

1. had to conceal information that is not conducive to the sales of your products under certain pressures.
2. had to highlight the advantages of your product or service and avoided the disadvantages under certain pressure.
3. had to weaken the shortcomings of the product or service for the benefit of your organization under certain pressures.
4. had to accept decisions that may offend the interests of others outside the organization.
5. had to exaggerate the truth to promote the cooperation between the partner and your organization.
6. had to depreciate competitors for the benefit of the organization under certain pressures.
7. had to turn a blind eye to our organization's behavior that offend the interests of others outside the organization.
8. had to conceal information from the public that could be damaging to your organization.
9. had to misrepresent the truth to make your organization look good.
10. had to misrepresent the truth to make your organization look good.
11. had to take some cheating measures to help the organization pass the external inspection.
12. had to exaggerate data or information that is good for the organization under certain pressures.
13. had to ignore the rights and interests of people outside the organization under certain pressures.
14. had to recommend customers to buy products that are not suitable for them to help your organization.
15. had to deflect or avoid topics that are not favorable to your organization.
16. had to withhold to customers the discounts he/she can get form your company.

Appendix D

Final UPB scales

INSTRUCTIONS: The following questions are about workplace behaviors you may have come across. In the last month, how often have you…

Compulsory UPB

Business-oriented compulsory UPB(CBOU)

1. had to highlight the advantages of your product or service and avoided the disadvantages under certain pressure.
2. had to weaken the shortcomings of the product or service for the benefit of your organization under certain pressures.
3. had to conceal information that is not conducive to the sales of your products under certain pressures.

Relationship-oriented compulsory UPB(CROU)

4. had to depreciate competitors for the benefit of the organization under certain pressures.
5. had to ignore the rights and interests of people outside the organization under certain pressures.
6. had to misrepresent the truth to make your organization look good.
7. had to take some cheating measures to help the organization pass the external inspection.

SCORING INSTRUCTIONS: Items are evaluated by a 7-point response scale (1=never, 2=once a month, 3=2-3 times a month, 4=once a week, 5=2-3 times a week, 6=once a day, 7= more than once a day).

Appendix E

Chinese Translation of the UPB scales

INSTRUCTIONS: 以下问题涉及你在工作场合中曾经遇到的行为。在过去的一个月中，你多经常…

Compulsory UPB

Business-oriented compulsory UPB(CBOU)

1. 迫于某种压力，不得不突出本单位产品或服务的优点而回避缺点。
2. 迫于某种压力，不得不为了本单位利益而弱化产品或服务的缺点。
3. 迫于某种压力，不得不隐瞒不利于本单位产品销售的信息。

Relationship-oriented compulsory UPB(CROU)

4. 迫于某种压力，不得不为了本单位利益而贬低竞争对手。
5. 迫于某种压力，不得不为了本单位利益而忽视组织外部人员的权益。
6. 不得不夸大事实使我的单位看起来更好。
7. 迫于某种压力，不得不通过作弊手段帮助单位通过外部检查。

评分说明：每个条目采用七点量表评分，1=从来没有，2=一个月一次，3=一个月 2 到 3 次，4=一周一次，5=一周 2 到 3 次，6=一天一次，7=一天超过一次）。
